# Polyketides from a soil -derived *Streptomyces* sp. CSDX076 and their antimicrobial activities

**DOI:** 10.7717/peerj.21830

**Published:** 2026-10-21

**Authors:** Shengyan Qian, Hengxing Cai, Afsar Khan, Bin Chen, Li Tuo, Guiguang Cheng

**Affiliations:** 1 Guizhou Provincial Key Laboratory of Microbial Resources and Drug Development, Zunyi Medical University, Zunyi, Guizhou, China; 2 School of Stomatology, Zunyi Medical University, Zunyi, China; 3 Shool of Pharmacy, Zunyi Medical University, Zunyi, China; 4 Department of Chemistry, COMSATS University Islamabad, Abbottabad, Pakistan; 5 School of Basic Medical Sciences, Zunyi Medical University, Zunyi, China; 6 Yunnan Institute of Food Safety, Kunming University of Science and Technology, Zunyi, China

**Keywords:** Streptomyces sp. CSDX076, Pseurotin A3-10 11-acetonide, Antimicrobial activity

## Abstract

A new naturally occurring polyketide, pseurotin A_3_-10,11-acetonide (1), along with two known analogs, pseurotin A (2) and pseurotin A_3_ (3), was isolated from the culture broth of* Streptomyces* sp. CSDX076. The structures of these compounds were determined by extensive 1D and 2D nuclear magnetic resonance (NMR), ultraviolet (UV), and high-resolution electrospray ionization mass spectrometry (HRESIMS) spectroscopic analyses. All isolated compounds (1-3) were evaluated for antimicrobial activity against a panel of seven pathogenic strains using disc diffusion and broth microdilution methods. Notably, compound 1 exhibited moderate activity against *Brucella melitensis* with a minimum inhibitory concentration (MIC) of 8.0 μg/mL, while compound 2 showed weak activity against *Staphylococcus epidermidis* (MIC = 128.0 μg/mL); compound 3 was inactive against all tested strains. This study reports, for the first time, the antibacterial effects of these compounds against the respective pathogens.

## Introduction

Pseurotin analogues are microbial polyketides, with unusual hetero-spirocyclic skeleton, which have gained considerable attention due to their unique structures and potent biological properties. The first member of this family, pseurotin A, was initially isolated from the fungus *Pseudeurotium ovalis* by Bloch and co-workers in 1976 and fully characterized in 1981 (Bloch et al., 1976; Bloch & Tamm, 1981). The bioactivities of pseurotins have been investigated primarily in *vitro* systems. Pseurotin A exhibited antimicrobial activity against *Bacillus subtilis*, *Escherichia coli*, *Pseudomonas aeruginosa*, and *Staphylococcus aureus* with minimum inhibitory concentration (MIC) values ranging from 15.62 to 31.25 μg/mL (Pinheiro et al., 2013). Pseurotin A was also reported to inhibit chitin synthase with an IC_50_ of 81 μM (Wenke, Anke & Sterner, 1993). Additionally, it showed cytotoxicity against glioma cell lines with IC_50_ values ranging from 0.51 to 29.3 μM (Anjum et al., 2018). Pseurotins A and D demonstrated anti-inflammatory effects by inhibiting nitric oxide production, iNOS expression, and IL-6 production in LPS-stimulated RAW 264.7 macrophages at concentrations up to 50 μM (Vasicek et al., 2020). Pseurotin A2 and azaspirofuran A showed antiseizure activity in the larval zebrafish PTZ seizure model (Copmans et al., 2018). Other reported activities include neuritogenic (Komagata et al., 1996) and antiallergic properties (Ishikawa et al., 2009). A number of pseurotin analogues have been isolated and characterized from *Aspergillus* sp. obtained from diverse sources, including the jellyfish *Nemopilema* spp. (Tao et al., 2016), the marine fish *Mugil cephalus* (Yamada et al., 2016), the medical plant *Bauhinia guianensis* (Pinheiro et al., 2013), the liverwort *Heteroscyphus tener* (Yuan et al., 2015), and marine sediments (Saraiva et al., 2015). *Aspergillus fumigatus* has proven to be a particularly prolific source of pseurotin analogues, with numerous derivatives (including pseurotins A, A_1_, A_2_, D, F1, 11-O-methylpseurotin A, 8-O-demethylpseurotin A, azaspirofurans A and B, cephalimysins M and N, and FD-838) reported from various strains of this species (Li et al., 2012; Yamada et al., 2016; Copmans et al., 2018; Wenke, Anke & Sterner, 1993; Xu et al., 2019). However, recent studies have demonstrated that the ability to produce pseurotin analogues is not restricted to fungi. Pseurotin A was first isolated from a *Streptomyces* species in our previous work (Qian et al., 2016). Subsequently, the same compound was also isolated from a marine *Bacillus* sp. (Anjum et al., 2018), and a new derivative, pseurotin G, was obtained from co-culture of *Streptomyces leeuwenhoekii* with *A. fumigatus* (Wakefield et al., 2017). These findings collectively indicate that pseurotin-type compounds can be produced by bacteria, a notion further supported by the present study.

In our ongoing search for microbial secondary metabolites with structural novelty and biological activity, a phytochemical investigation on the fermentation of *Streptomyces* sp. CSDX076 was conducted. Our previous study on this strain resulted in the isolation of only one pseurotin-type compound from 17 g of crude extract, while other minor components could not be characterized at that time (Qian et al., 2016). In the present study, although a smaller amount of crude extract (12 g) was obtained from the same scale of fermentation, improved technical proficiency in separation using the same methods (repeated normal-phase silica gel column chromatography combined with reversed-phase Medium-pressure liquid chromatography (MPLC)) enabled the successful isolation of three pseurotin derivatives, including one new natural product, pseurotin A3-10,11-acetonide (**1**) , together with two known compounds. The structures of these compounds were determined by means of 1D and 2D nuclear magnetic resonance (NMR) spectroscopic techniques, and by comparison with those reported in the literature. These compounds were evaluated for antimicrobial activities against a panel of seven pathogenic strains (listed in Materials and Methods). Herein, we report the isolation, structure elucidation, and antimicrobial activities of these compounds.

## Materials & Methods

### General experimental procedures

All solvents and reagents were of analytical grade and obtained from Chengdu Kelong Chemical Co., Ltd. (Sichuan, China). MPLC was carried out on a Büchi pump system equipped with glass columns (15 × 230 and 26 × 460 mm) packed with C18 silica gel. Silica gel (200–300 mesh; Qingdao Marine Chemical Ltd., Qingdao, China) and Chromatorex C18 (20–45 µm; Fuji Silysia Chemical Ltd., Tokyo, Japan) were used for column chromatography. Detailed parameters for ultraviolet (UV), NMR, infrared (IR), and ultra-high performance liquid chromatography coupled with high-resolution mass spectrometry (UHPLC-HRMS) analyses are provided in the Analytical methods subsection below. All spectroscopic and spectrometric measurements were performed at the Analytical and Testing Center of Kunming Institute of Botany, Chinese Academy of Sciences (Kunming, China).

### Bacterial material

The bacterial strain designated CSDX076 was isolated from soil collected in Chishuidanxia, Guizhou Province, China, in October 2012. It was identified as a member of the genus *Streptomyces* based on morphological observation (aerial mycelium and spiral spore chains on GYM agar) and chemotaxonomic analysis (presence of LL-diaminopimelic acid in cell wall, characteristic of chemotype I of the genus *Streptomyces*). Prior to fermentation, the strain was authenticated by Professor Tuo (co-author of this paper), a microbiologist specializing in actinomycete taxonomy and natural product discovery at the Life Sciences Institute, Zunyi Medical University. The strain has been deposited at the China Center for Type Culture Collection (CCTCC) (Wuhan University, Wuhan, China) under the accession number CCTCC M 2014241. The same strain was previously used in our studies on secondary metabolite production (Qian et al., 2016; Qian et al., 2018).

### Fermentation, extraction and isolation

A stock culture of *Streptomyces* sp. CSDX076 strain was maintained at 4 °C on Glucose-Yeast Extract-Malt Extract (GYM) agar slant consisting of glucose four g, malt extract four g, yeast extract 10 g, calcium carbonate two g and microelement mixed liquid 0.5 mL in 1 L distilled water (pH 8.0). The stock culture was inoculated into 500 mL Erlenmeyer flask containing 200 mL of seed medium which was same as above without agar. The flask culture was incubated on a rotary shake (160 rpm) at 28 °C for 72 h. Two hundred milliliters of the flask culture were transferred to 5,000 mL Erlenmeyer flask containing 1,000 mL of the producing medium, which contained the same ingredients as seed medium. The fermentation was carried out at 28 °C for 120 h on a rotary shaker (160 rpm).

The whole fermented cultures (50 L) were extracted with ethyl acetate (EtOAc) twice at room temperature for 24 h on a rotary shaker (70 rpm). The combined EtOAc extracts were concentrated under reduced pressure to give a crude extract (12 g) for further separation.

The crude extract (12 g) was fractionated by silica gel column chromatography (5 × 60 cm, 200–300 mesh) eluted with a gradient of CHCl_3_-acetone (30:1 → 20:1 → 10:1 → 5:1 → 2:1, *v*/*v*) under gravity flow. Fractions of 250 mL were collected and concentrated under reduced pressure using a rotary evaporator, then each residue was dissolved in acetone and transferred to 25 mL vials. These concentrated fractions were monitored by thin-layer chromatography (TLC) on silica gel GF_2__5__4_ plates using various solvent systems, including CHCl_3_-acetone (5:1), CHCl_3_-methanol (10:1), and petroleum ether-acetone (1:1), to ensure comprehensive detection of components with different polarities. Spots were visualized under UV light (254 and 365 nm) and by spraying with 10% H_2_SO_4_ in ethanol followed by heating. Based on TLC pattern similarity across these systems, fractions were pooled into six major fractions (Fr. 1–Fr. 6).

The six fractions (Fr. 1-Fr. 6) were evaluated by TLC using CHCl_3_-acetone (5:1) as the developing solvent. Fr. 1 and Fr. 2 showed weak spots near the solvent front (retention factor (Rf) > 0.8) with limited material; Fr. 1 was not pursued further due to insufficient quantity. Fr. 3 showed a major spot at retention factor (Rf) 0.75. Fr. 5 showed a major spot at retention factor (Rf) 0.30. Fr. 6 remained at the origin (retention factor (Rf) 0.0), indicating highly polar components. Compounds from Fr. 2, 3, 5, and 6 were identified as known compounds and are not part of the present study. In contrast, Fr. 4 displayed a major spot at retention factor (Rf) 0.4 along with several minor spots, suggesting the presence of diverse metabolites and making it the most promising fraction for further purification. No authentic standard was used at this stage, as the target compounds were unknown, which is standard practice in natural product isolation. Therefore, Fr. 4 (1.2 g) was selected for further purification.

Fr. 4 (1.2 g) was subjected to reversed-phase C_18_ MPLC (Büchi pump system, 3 × 40 cm column, 20–45 µm) eluted with a stepwise gradient of CH_3_OH-H_2_O (30:70 → 50:50 → 70:30 → 80:20, *v*/*v*) at a flow rate of 20 mL/min. Fractions of 200 mL were collected, concentrated under reduced pressure, and the residue of each fraction was dissolved in methanol and transferred to 25 mL vials. These fractions were monitored by TLC using three solvent systems: petroleum ether-acetone (1:1), CHCl_3_-acetone (5:1), and CHCl_3_-methanol (12:1), to ensure comprehensive detection of components with different polarities. Based on TLC pattern similarity, fractions were combined into four subfractions (Fr. 4.1–Fr. 4.4).

Fr. 4.2 (196 mg) was chromatographed over a silica gel column, eluted with petroleum ether-acetone (4:1 to 2:1) to give compound **1** (120 mg).

Fr.4.3 (128 mg) was chromatographed over a RP-18 silica gel column, eluted with CH_3_OH-H_2_O (20:80 to 50:50) to give compound **2** (45 mg) and compound **3** (9.8 mg).

### Analytical methods

**UV spectrophotometry:** The UV spectrum was recorded on a Shimadzu UV-2401A spectrophotometer (Shimadzu Corp., Kyoto, Japan) using a one cm quartz cuvette. The sample was dissolved in methanol (approx. 0.1 mg/mL) and scanned from 200 to 400 nm at a speed of 200 nm/min. Baseline correction was performed with pure methanol.

**IR spectroscopy:** The IR spectrum was recorded on a Bruker FT-IR Tensor 27 spectrometer (Bruker Corp., Billerica, MA, USA) with potassium bromide (KBr) pellets. The spectrum was measured in the range of 4000–400 cm^−^^1^ at a resolution of four cm^−^^1^, with 16 scans co-added. Background was subtracted using a pure KBr pellet.

**MR spectroscopy** NMR spectra were acquired on a Bruker DRX-500 MHz spectrometer (Bruker Corp., Billerica, MA, USA). The sample was dissolved in acetone-*d*_6_ with tetramethylsilane (TMS) as the internal standard (*δ* 0.00). ^1^H NMR spectrum was recorded at 500 MHz using a 30° pulse sequence (zg30), 64 scans, a relaxation delay of 2 s, and a spectral width of 10 kHz. ^1^^3^C NMR spectrum was recorded at 125 MHz with broadband proton decoupling (zgpg30), 1,024 scans, and a relaxation delay of 2 s. Chemical shifts (*δ*) are reported in parts per million (ppm), and coupling constants (*J*) are given in Hertz (Hz)**.**

**UHPLC-HRMS:** UHPLC-HRMS data were obtained on a Thermo Ultimate 3000 ultra-high performance liquid chromatography (UHPLC) system (Thermo Fisher Scientific, Waltham, MA, USA) coupled to an electrospray ionization (ESI) mass spectrometer. Chromatographic separation was performed on a C_18_ column (2.1 × 100 mm, 1.9 µm) maintained at 40 °C. The mobile phase consisted of (A) 0.1% formic acid in water and (B) acetonitrile. The gradient program was: 0–2 min, 5% B; 2–15 min, 5–95% B; 15–18 min, 95% B; followed by re-equilibration for 3 min. The flow rate was 0.3 mL/min, and the injection volume was two µL. The ESI source was operated in positive ion mode with the following parameters: spray voltage 3.5 kV, sheath gas flow 40 arbitrary units, auxiliary gas flow 10 arbitrary units, and capillary temperature 320 °C. Full-scan high-resolution mass spectrometry (HRMS) data were acquired at a resolution of 70,000 (full width at half maximum (FWHM) at m/z 200) over the mass range m/z 100–1500. Data acquisition and processing were performed using Xcalibur software (Thermo Fisher Scientific).

### Antibacterial assay

### Test microorganisms

The following strains were used for antimicrobial testing: methicillin-resistant *Staphylococcus aureus* (MRSA, ATCC 43300), *Staphylococcus epidermidis* (ATCC 12228), *Bacillus subtilis* (ATCC 6633), *Brucella melitensis* (ATCC 23456), extended-spectrum *β*-lactamase-producing *Escherichia coli* (ESBLEC, ATCC 35218), *Proteus vulgaris* (ATCC 13315), and *Candida albicans* (ATCC 10231). All strains were obtained from the American Type Culture Collection (ATCC, Manassas, VA, USA).

### Disc diffusion assay (preliminary screening)

The disc diffusion test was performed as described by Bauer et al. (1966) for preliminary screening. Compounds **1**-**3** were dissolved in dimethyl sulfoxide (DMSO) to a concentration of three mg/mL. Sterile filter paper discs (six mm diameter) were impregnated with 10 μL of each solution (30 µg per disc) and placed on Mueller-Hinton agar plates that had been inoculated with 0.5 McFarland standard suspensions of the test strains (listed in ‘Test microorganisms’). As a negative control, discs were impregnated with 10 μL of DMSO (the same solvent and volume used for the test compounds) to ensure that any observed inhibition was due to the compounds themselves and not the solvent. The plates were incubated at 37 °C for 24 h, after which the diameters of the inhibition zones (including the six mm disc) were measured in millimeters. This assay was conducted as a preliminary screening; the antibacterial activity was subsequently confirmed by quantitative MIC determination using the broth microdilution method.

### Determination of minimum inhibitory concentration–broth microdilution method

Based on the preliminary disc diffusion results, compounds that showed inhibitory activity were further evaluated for their MIC values using the broth microdilution method in 96-well microtiter plates according to the Clinical and Laboratory Standards Institute (CLSI) guidelines (M07-A11). Compounds were dissolved in DMSO and then diluted with Mueller-Hinton broth (MHB) to prepare two-fold serial dilutions. For compound **1**, the final concentration range tested was 1–512 μg/mL against *B. melitensis*; for compound **2**, the same concentration range (1–512 μg/mL) was tested against *S. epidermidis*. Each well contained 100 μL of the compound dilution and 100 μL of bacterial suspension, adjusted to a final inoculum of approximately 5 × 10^5^ CFU/mL. MHB with 1% DMSO served as a negative control. The plates were incubated at 37 ^∘^C for 18–20 h. The MIC was defined as the lowest concentration of the compound that completely inhibited visible bacterial growth.

## Results

### Structure elucidation

Compound **1** (see Fig. 1), obtained as a white amorphous powder, was assigned a molecular formula of C_25_H_29_NO_8_ by the molecular ion peak at m/z 472.1949 [M+H]^+^ in its high-resolution electrospray ionization mass spectrometry (HRESIMS) (see Fig. S1), indicating 12 degrees of unsaturation. The UV (see Fig. S2) absorption maximum at 206 nm indicated the presence of benzene ring, while the IR (see Fig. S3) absorption bands at 3274, 1739, 1704, and 1632 and 1594 cm^−1^ respectively revealed the hydroxy group, carbonyl group, and benzene ring functionalities. The ^1^H NMR spectrum (see Fig. S4) showed characteristic signals for monosubstituted benzene ring at *δ*_H_ 8.32 (2H, t, *J* = 7.6 Hz), 7.51 (2H, t, *J* = 7.6 Hz), and 7.67 (1H, t, *J* = 7.6 Hz). An amino proton signal at *δ*_H_ 9.03 (s, 1H), four signals for methyls at *δ*_H_ 1.63 (s, 3H), 1.46 (s, 3H), 1.40 (s, 3H), and 0.91 (t, *J* = 7.5 Hz, 3H), and one methoxy signal at *δ*_H_ 3.38 (s, 3H) were also observed. Two downfield olefinic proton signals at *δ*_H_ 5.66 (1H, dd, *J* = 11.0, 9.0 Hz) and 5.58 (1H, dt, *J* = 11.0, 7.5 Hz) confirmed the presence of a cis double bond. The ^13^C NMR data of compound **1** coupled with distortionless enhancement by polarization transfer (DEPT) (see Fig. S5) and heteronuclear single quantum coherence (HSQC) spectra (see Fig. S6) showed 25 carbon resonances, assigned to one methoxy, four methyls, one methylene, ten methines (three oxygenated and seven olefinic), six quaternary carbons (three oxygenated and three olefinic), and three carbonyl carbons. The aforementioned data were closely related to those of pseurotin A (Bloch & Tamm, 1981), suggesting compound **1** was a pseurotin-type alkaloid. The main difference was the presence of three additional carbon signals at *δ*_C_ 25.2 (q), 26.7 (q), 111.1 (s). The deshielded signal at *δ*_C_ 111.1 was assigned to a di-oxygenated carbon (C-25). Besides three carbonyls, a benzene ring, and a C-C double bond, the degrees of unsaturation required three rings to meet the molecular formula, indicating that **1** must possess one additional ring. For the NMR data of compound **1**, see Table 1.

**Figure 1:**
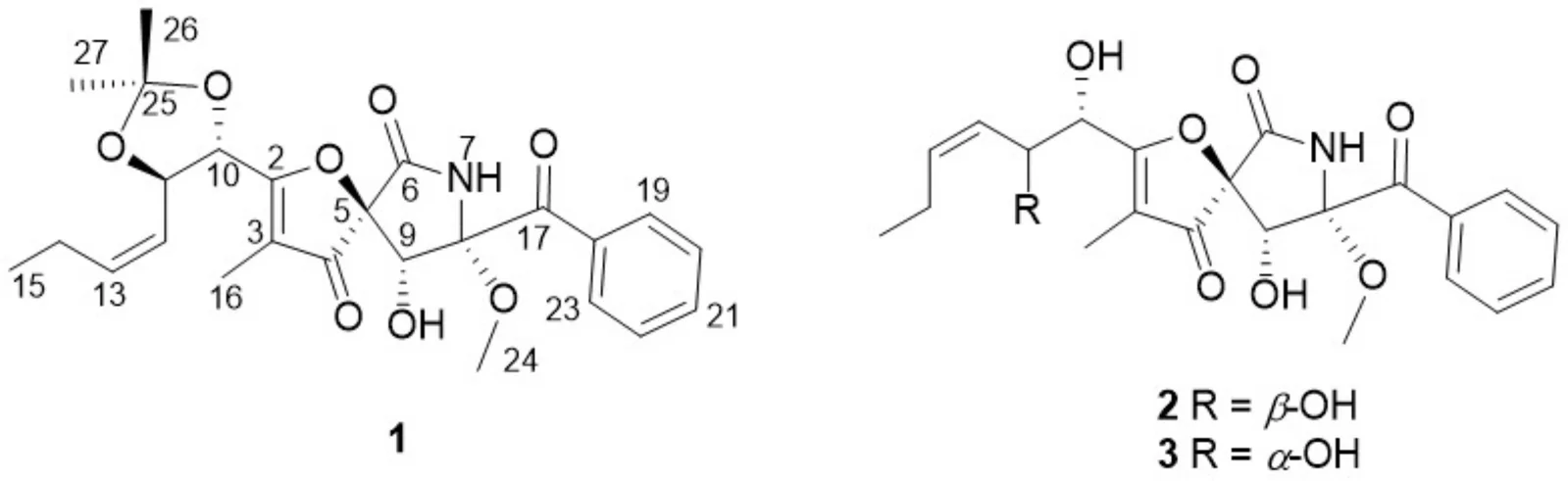
Chemical structures of compounds 1–3.

**Table 1:** ^1^H and ^13^C NMR spectroscopic data of compound **1** in acetone-*d*_6_.

Position	*δ*_C_	*δ*_H_mult. (*J* in Hz)
2	166.6, C	
3	113.0, C	
4	184.4, CO	
5	92.8, C	
6	197.7, CO	
8	92.4, C	
9	75.9, CH	4.61, d (11.0)
10	74.6, CH	5.37, d (7.2)
11	74.7, CH	5.27, d (7.2)
12	124.9, CH	5.66, dd (11.0, 9.0)
13	137.8, CH	5.58, dt (11.0, 7.5)
14	21.5, CH_2_	2.08-2.17, m
15	14.6, CH_3_	0.91, t (7.5)
16	5.6, CH_3_	1.63, s
17	196.4, CO	
18	134.5, C	
19	131.3, CH	8.32, t (7.6)
20	129.2, CH	7.51, t (7.6)
21	134.7, CH	7.67, t (7.6)
22	129.2, CH	7.51, t (7.6)
23	131.3, CH	8.32, t (7.6)
24	52.1, OCH_3_	3.38, s
25	111.1, C	
26	25.2, CH_3_	1.40, s
27	26.7, CH_3_	1.46, s
NH		9.03, s

**Notes.**

Data were measured at 500 MHz for ^1^H NMR spectrum and 125 MHz for ^13^C NMR spectrum.

In the heteronuclear multiple bond correlation (HMBC) spectrum (see Fig. S7), the correlations of *δ*_H_ 5.27 (1H, d, *J* = 7.2 Hz, H-11) with *δ*_C_ 124.9 (d, C-12), 111.1 (s, C-25), and 74.6 (d, C-10); of *δ*_H_ 5.37 (1H, d, *J* = 7.2 Hz, H-10) with *δ*_C_ 111.1 (s, C-25) and 74.7 (d, C-11); and of 1.46 (s, 3H, H-27) and 1.40 (s, 3H, H-26) with 111.1 (s, C-25) established the presence of a five-membered ring in **1**. For the key HMBC and ^1^H-^1^H COSY correlations of compound **1**, see Fig. 2. Detailed analysis of 2D NMR spectroscopic data of compound **1** revealed that the other parts were the same to those of pseurotin A. Moreover, rotating-frame overhauser effect spectroscopy (see Fig. S9) correlations indicated that the relative configuration of compound **1** was the same as that of pseurotin A (Bloch & Tamm, 1981). In addition, the optical rotation value of compound **1** [+ 72.7 (c 0.10, Methanol (MeOH))] was similar in magnitude and with the same sign to that of acetyl derivative of pseurotin A3, the absolute configuration of which was determined by quantum chemical calculations through optical rotation, electronic circular dichroism (ECD), and vibrational circular dichroism (VCD) and comparison with experimental values (Xu et al., 2016). Therefore, compound **1** was determined as shown, and was named pseurotin A3-10,11-acetonide.

**Figure 2:**
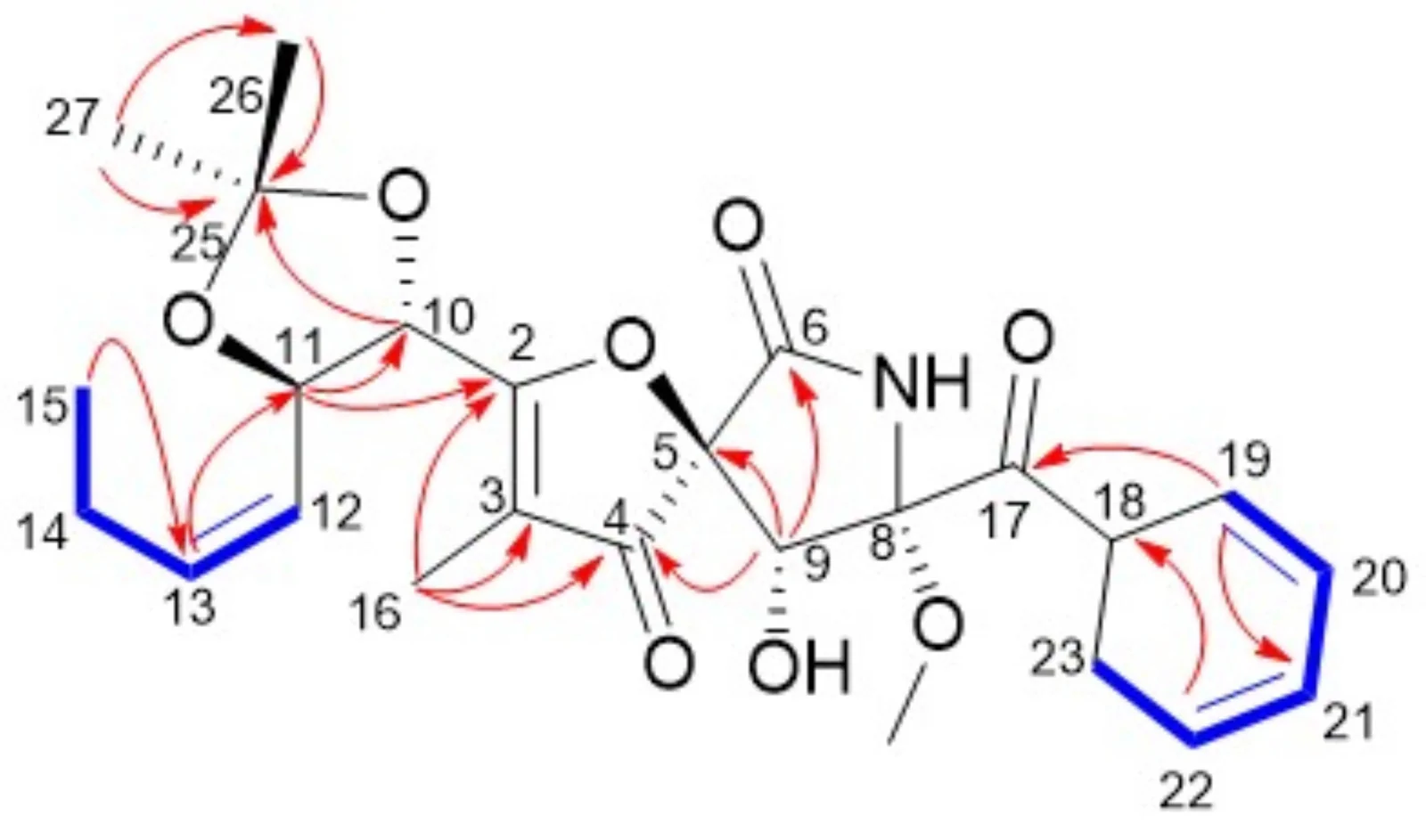
Selected HMBC (→) and ^1^H-^1^H COSY (–) correlations of compound 1.

Two pseurotin derivatives were also obtained from cultured broth of *Streptomyces* sp. CSDX076. Their structures were identified as pseurotin A (**2**) and pseurotin A3 (**3**) by comparison of their spectroscopic data with those reported in the literature (Bloch & Tamm, 1981; Xu et al., 2016).

Pseurotin A_3_-10,11-acetonide (**1**): White amorphous powder; [*α*]28.8 D = +72.7 (*c* = 0.10, CH_3_OH); UV (MeOH) *λ*_max_ (log *ɛ*) 206 (2.59) nm; IR (KBr): *ν*_max_ 3274, 2964, 2934, 2872, 1739, 1704, 1632, 1594, 1493, 1460, 1382, 1190, 1160, 1082, 1053, 1062, 903, 876, and 667 cm^−1^; ^1^H (Acetone-*d*_6_, 500 MHz) and ^13^C NMR (Acetone-*d*_6_, 125 MHz) data, see Table 1; HRESIMS m/z 472.1949 [M + H]^+^ (calcd. for C_25_H_30_NO_8_, 472.1966).

Pseurotin A (**2**): White amorphous powder; ^1^H (Acetone-*d*_6_, 500 MHz) *δ*_H_ 9.17 (1H, s, -NH), 8.34 (2H, d, *J* = 8.8 Hz, H-19, 23), 7.67 (1H, t, *J* = 8.9 Hz, H-21), 7.52 (2H, t, *J* = 9.4 Hz, H-20, 22), 5.52 (1H, dd, *J* = 9.0, 5.0 Hz, H-13), 5.48 (1H, t, *J* = 10.0 Hz, H-12), 4.68 (1H, m, H-11), 4.63 (1H, d, *J* = 4.6 Hz, H-10), 4.55 (1H, s, H-9), 3.38 (3H, s, 8-OCH_3_), 2.10−2.16 (2H, sext, *J* = 8.8 Hz, H-14), 1.71 (3H, s, H-16), 0.91 (3H, t, *J* = 9.0 Hz, H-15)_°_^13^C-NMR (125M Hz, acetone-*d*_6_) *δ*_C_ 197.8 (s, C-4), 196.4 (s, C-17), 187.6 (s, C-6), 167.3 (s, C-2), 135.7 (d, C-13), 134.7 (d, C-21), 134.5 (s, C-18), 131.3 × 2 (d, C-19, 23), 129.2 (d, C-12), 129.2 × 2 (d, C-20, 22), 113.6 (s, C-3), 92.6 (s, C-8), 92.5 (s, C-5), 75.3 (d, C-9), 72.8 (d, C-11), 69.6 (d, C-10), 52.5 (q, 8-OCH_3_), 21.8 (t, C-14), 14.5 (q, C-15), 5.8 (q, C-16).

Pseurotin A_3_ (**3**): White amorphous powder; ^1^H (Acetone-*d*_6_, 500 MHz) ^1^H-NMR (500 MHz, acetone-*d*_6_) *δ*_H_ 9.03 (1H, s, -NH), 8.20 (2H, d, *J* = 10.1 Hz, H-19, 23), 7.63 (1H, t, *J* = 8.8 Hz, H-21), 7.51 (2H, t, *J* = 9.3 Hz, H-20, 22), 5.54 (1H, dd, *J* = 9.2, 5.5 Hz, H-13), 5.45 (1H, m, H-12), 4.83 (1H, d, *J* = 4.6 Hz, H-10), 4.82 (1H, m, H-9), 4.63 (1H, s, H-11), 3.25 (3H, s, 8-OCH_3_), 2.09–2.16 (2H, m, H-14), 1.73 (3H, s, H-16), 0.93 (3H, t, *J* = 9.0 Hz, H-15)° ^13^C-NMR (125M Hz, acetone-*d*_6_) *δ*_C_ 199.6 (s, C-4), 194.6 (s, C-17), 186.7 (s, C-6), 168.9 (s, C-2), 135.9 (d, C-13), 135.8 (d, C-21), 134.0 (s, C-18), 130.6 × 2 (d, C-19, 23), 129.6 (d, C-12), 129.1 × 2 (d, C-20, 22), 113.1 (s, C-3), 97.6 (s, C-8), 89.5 (s, C-5), 77.1 (d, C-9), 72.9 (d, C-11), 69.8 (d, C-10), 51.7 (q, 8-OCH_3_), 21.9 (t, C-14), 14.5 (q, C-15), 5.8 (q, C-16).

### Antibacterial activity

The isolated compounds were evaluated for their antimicrobial activity against a panel of seven pathogenic strains (listed in ‘Test microorganisms’). Initial screening was performed using the Kirby-Bauer disk diffusion assay at a concentration of 30 µg per disc (10 μL of a 3 mg/mL solution in DMSO). The results indicated that pseurotin A_3_-10,11-acetonide (**1**) exhibited the most potent activity, displaying a significant zone of inhibition (33 mm) against *B. melitensis*. In contrast, pseurotin A (**2**) showed selective activity against *S. epidermidis*, with a 13 mm inhibition zone (see Table 2). Compound **3** showed no inhibitory activity against any of the tested strains at the same concentration.

**Table 2:** Antimicrobial activity of compounds 1–3 as inhibition zone diameters (mm).

Strains	Zone of inhibition (diameter in mm)		
	Pseurotin A_3_-10,11- acetonide	Pseurotin A	Pseurotin A_3_
Gram positive bacteria			
MRSA	–	–	–
S. e.	–	13	–
B. s.	–	–	–
Gram negative bacteria			
P. v.	–	–	–
B. m.	33	–	–
ESBLEC	–	–	–
*Fungus*			
C. a.	–	–	–

**Notes.**

‘–’ indicates no zone of inhibition; MRSA, methicillin-resistant *Staphylococcus aureus*; S. e., *Staphylococcus epidermidis*; B. s., *Bacillus subtilis*; P. v., *Proteus vulgaris*; B. m., *Brucella melitensis*; ESBLEC, Extended spectrum β-lactamase producing *Escherichia coli*, C. a., *Candida albicans*.

To quantify the antibacterial potency, the MIC of the active compounds was determined using the broth microdilution method. Consistent with the disk diffusion results, compound **1** demonstrated strong activity against *B. melitensis* with an MIC value of 8.0 μg/mL. Compound **2** was active against *S. epidermidis* with an MIC of 128.0 μg/mL (see Table 3). The remaining tested compounds (**3**) and other microbial strains showed no appreciable activity in these assays.

**Table 3:** MIC values of compounds 1 and 2 against selected bacterial strains.

Strains	MIC (µg/mL)	
	Pseurotin A_3_-10,11-acetonide	Pseurotin A
S. e.	–	128.0
B. m.	8.0	–

**Notes.**

‘–’ indicates no zone of inhibition; S. e., *Staphylococcus epidermidis*; B. m., *Brucella melitensis*

## Discussion

In the present study, we report the isolation of three pseurotin derivatives from *Streptomyces* sp. CSDX076, including a new compound (**1**) and pseurotin A_3_ (**3**) reported from a *Streptomyces* source for the first time. This finding further expands the known taxonomic distribution of pseurotin-type metabolites, which have been predominantly documented from fungi (Bloch & Tamm, 1981; Mehedi et al., 2010; Pinheiro et al., 2013). Moreover, our antimicrobial evaluation revealed previously undocumented activities: compound **1** (pseurotin A3 10,11 acetonide) exhibited potent and selective activity against *Brucella melitensis* (MIC = 8.0 μg/mL), a category B biothreat agent and the causative pathogen of brucellosis, while compound **2** (pseurotin A) showed weak activity against *Staphylococcus epidermidis* (MIC = 128.0 μg/mL). These findings not only demonstrate that *Streptomyces* can serve as an alternative source of pseurotin type compounds but also highlight the potential of pseurotin derivatives as selective antibacterial agents against clinically relevant pathogens.

The antibacterial evaluation revealed distinct selectivity profiles among the isolated compounds. Compound **1** exhibited potent activity against *B. melitensis* with an MIC of 8.0 μg/mL, while showing no activity against the other tested strains at concentrations up to 128 μg/mL. In contrast, compound **2** displayed weak activity against *S. epidermidis* (MIC = 128.0 μg/mL) and was inactive against the remaining strains. Compound **3** showed no antibacterial activity against any of the tested pathogens. To the best of our knowledge, this is the first report of antibacterial activity of any pseurotin type compound against *B. melitensis*. The selective activity of compound **1** against this pathogen is particularly significant, as brucellosis remains a major zoonotic disease with limited therapeutic options, especially in developing countries (Pappas et al., 2006). The enhanced activity of compound **1** compared to compound **2** may be attributed to the presence of the acetonide moiety at C-10 and C-11, which appears to confer improved specificity toward *B. melitensis*.

While direct comparison with other pseurotin analogues is limited by the absence of literature data on the same strains, previous studies have reported that pseurotin A exhibits activity against other Gram positive bacteria, including *Staphylococcus aureus* (MIC = 15.62 μg/mL; Pinheiro et al., 2013). Our results showed that pseurotin A (compound **2**) was considerably less potent against *S. epidermidis* (MIC = 128.0 μg/mL) than against *S. aureus* (MIC = 15.62 μg/mL), indicating that the antibacterial spectrum of pseurotin A may be strain dependent even within the same genus. The structural novelty of compound **1**, combined with its selective antibacterial activity, provides a promising starting point for further structure activity relationship studies and the development of new therapeutic agents against brucellosis.

The biosynthetic origin of pseurotin analogues has been extensively characterized in fungi. In *Aspergillus fumigatus*, the pseurotin biosynthetic gene cluster (BGC) contains a hybrid polyketide synthase–nonribosomal peptide synthetase (PKS–NRPS) gene, *psoA*, which is essential for the formation of the pseurotin core structure (Maiya et al., 2007). The cluster also encodes tailoring enzymes such as PsoF, a bifunctional epoxidase–C methyltransferase that contributes to the structural diversity of pseurotin analogues (Tsunematsu et al., 2014). A remarkable feature of the pseurotin BGC in *A. fumigatus* is its organization within an intertwined secondary metabolite supercluster, where the genes for fumagillin and pseurotin are physically intermingled under the control of the master regulator LaeA (Wiemann et al., 2013).

In contrast to the well characterized fungal pseurotin BGC, the genetic basis for pseurotin production in bacteria remains largely unexplored. To date, no pseurotin BGC has been conclusively identified in *Streptomyces* or other bacterial genera. However, the isolation of pseurotin A from *Streptomyces* sp. CSDX076 and a marine *Bacillus* sp. (Anjum et al., 2018), along with the production of pseurotin G through co cultivation of *Streptomyces leeuwenhoekii* with *A. fumigatus* (Wakefield et al., 2017), strongly suggests that bacteria possess the genetic capacity to produce these compounds. In the co-cultivation study, the pseurotin G gene cluster was identified through genome scanning of *S. leeuwenhoekii*, but its expression was only triggered upon fungal–bacterial interaction, indicating that the bacterial BGC may be cryptic under standard laboratory conditions. The presence of pseurotin biosynthetic capacity in both fungi and bacteria raises intriguing questions about the evolutionary origin and potential horizontal transfer of the relevant gene clusters. Future work will focus on genome sequencing of *Streptomyces* sp. CSDX076 to identify the putative BGC responsible for pseurotin production in this strain and to explore its regulation and biotechnological potential.

## Conclusions

In conclusion, three pseurotin derivatives were successfully isolated from the cultured broth of *Streptomyces* sp. CSDX076. Among them, pseurotin A3-10,11-acetonide (**1**) is reported as a new natural product and compound **3** (pseurotin A3) is reported from a *Streptomyces* source for the first time. Antimicrobial assays demonstrated that compound **1** possesses moderate activity against *Brucella melitensis* (MIC = 8.0 µg/mL), while compound **2** showed weak active against *Staphylococcus epidermidis* (MIC = 128.0 µg/mL); compound 3 was inactive against all tested strains. To the best of our knowledge, this is the first report of antibacterial activity of pseurotin-type compounds against these pathogens. These findings not only expand the chemical diversity and biological source of the pseurotin family but also identify new, structure-dependent antibacterial profiles worthy of further investigation.

## Supplemental Information

10.7717/peerj.21830/supp-1 Supplemental Information 1 Processed 2D NMR spectra (HSQC, HMBC, COSY) in TIFF format for the new compounds, with all key correlations clearly annotated

10.7717/peerj.21830/supp-2 Supplemental Information 2 Raw data Complete raw FID files for all 1D NMR experiments (1H, 13C, DEPT) for compounds 1–3, acquired on a Bruker spectrometer. These are the original, unprocessed instrument files in machine-readable format.

## Additional Information and Declarations

### Data Availability

The following information was supplied regarding data availability: The data is available in the Supplemental Files.
