# Supplementary material for: Polyketides from a soil -derived Streptomyces sp. CSDX076 and their antimicrobial activities
Source: PeerJ. 2026 Oct 21;14:e21830. doi: 10.7717/peerj.21830 (PMC13695420; doi:10.7717/peerj.21830)
Supplement: Supplementary file 1 [file peerj-14-21830-s001.docx]

**Contents**

Figure S1. HRESI spectrum of compound **1**

Figure S2. UV spectrum of compound **1**

Figure S3. IR spectrum of compound **1**

Figure S4. ^1^H NMR spectrum of compound **1**

Figure S5. ^13^C NMR and DEPT spectrum of compound **1**

Figure S6. HSQC spectrum of compound **1**

Figure S7. HMBC spectrum of compound **1**

Figure S8. ^1^H-^1^H COSY spectrum of compound **1**

Figure S9. ROESY spectrum of compound **1**

Figure S10. ^1^H NMR spectrum of compound **2**

Figure S11. ^13^C NMR and DEPT spectrum of compound **2**

Figure S12. ^1^H NMR spectrum of compound **3**

Figure S13. ^13^C NMR and DEPT spectrum of compound **3**

Figure S15. The minimum inhibitory concentration (MIC, µg/mL) of compound **1** against *Bacterium burger*

Figure S16. The minimum inhibitory concentration (MIC, µg/mL) of compound **2** against *Staphylococcus epidermidis*

Figure S1. HRESI spectrum of compound **1**

Figure S2. UV spectrum of compound **1**

Figure S3. IR spectrum of compound **1**

Figure S4. ^1^H NMR spectrum of compound **1**

Figure S5. ^13^C NMR and DEPT spectrum of compound **1**

Figure S6. HSQC spectrum of compound **1**

Figure S7. HMBC spectrum of compound **1**

Figure S8. ^1^H-^1^H COSY spectrum of compound **1**

Figure S9. ROESY spectrum of compound **1**

Figure S10. ^1^H NMR spectrum of compound **2**

Figure S11. ^13^C NMR spectrum of compound **2**

Figure S12. ^1^H NMR spectrum of compound **3**

Figure S13. ^13^C NMR and DEPT spectrum of compound **3**

Figure S15. The minimum inhibitory concentration (MIC, µg/mL) of compound **1** against *Bacterium burger*

Figure S16. The minimum inhibitory concentration (MIC, µg/mL) of compound **2** against *Staphylococcus epidermidis*
